# Intravenous thrombolysis in the context of stroke and cancer

**DOI:** 10.1007/s11239-025-03156-5

**Published:** 2025-07-17

**Authors:** Alessandra Burini, Maurizio Paciaroni, Lucio D’Anna, Fedra Kuris, Valentina Maniaci, Mariarosaria Valente, Gian Luigi Gigli, Giovanni Merlino

**Affiliations:** 1 Clinical Neurology, Department of Medicine (DMED), University of Udine, Udine, Italy; 2 Clinical Neurology, Department of Head-Neck and Neuroscience, Azienda Sanitaria Universitaria Friuli Centrale (ASU FC), Udine, Italy; 3 Department of Neuroscience and Rehabilitation, University of Ferrara, Ferrara, Italy; 4 Department of Brain Sciences, Imperial College London, London, UK; 5 Department of Stroke and Neuroscience, Charing Cross Hospital, Imperial College Healthcare NHS Trust, London, UK; 6 SOC Oncology, Azienda Sanitaria Universitaria Giuliano-Isontina, Gorizia and Monfalcone Hospital, Gorizia, Italy; 7 SOSD Stroke Unit, Department of Head-Neck and Neuroscience, Azienda Sanitaria Universitaria Friuli Centrale (ASU FC), Udine, Italy

**Keywords:** Stroke, Cancer, Intravenous thrombolysis, Stroke management, Guidelines, Best practice

## Abstract

**Background:**

Cancer patients are at an increased risk for ischemic and hemorrhagic strokes. Ischemic stroke in this population often presents with distinctive features, such as cryptogenic etiology and multiple ischemic lesions, and is driven by cancer-associated coagulopathy, complicating management strategies.

**Methods:**

We reviewed current literature on intravenous thrombolysis (IVT) for acute ischemic stroke in cancer patients through PubMed search with no time limits. We included international guidelines, meta-analyses, cohort studies, and case series to evaluate its safety and efficacy. This descriptive review aims to evaluate the risks and benefits of thrombolytic treatment in patients with acute stroke and cancer.

**Discussion:**

Despite limited high-quality evidence (no randomized trial), studies suggest that IVT is generally safe and effective in cancer patients with ischemic stroke. However, treatment should be individualized, considering specific contraindications and the patient’s tumor characteristics. The 2019 American Heart Association/American Stroke Association guidelines contraindicate IVT in patients with gastrointestinal or intra-axial tumors; conversely, these conditions are not explicitly mentioned in the 2021 European Stroke Organization guidelines, as recent studies have not proven them to be at higher risk per se. Particular attention should be given to coagulation abnormalities, recent surgery, and concomitant medications. Thus, cautious and multidisciplinary management is needed. Further research is essential to define risk stratification for this complex population better. Multicentered, well-designed prospective studies are crucial and should also differentiate patients based on tumor site, histology, and molecular characteristics that could impact both thrombotic and hemorrhagic risk.

## Introduction

The global burden of cancer is rising due to several factors, including an aging population, heightened risk factors, earlier diagnoses, and more effective therapies [1, 2]. As a result, cancer patients experience longer survival and improved quality of life, highlighting the need for tailored approaches to managing comorbid conditions.

Both ischemic and hemorrhagic strokes are more common in patients with active cancer than in those without cancer, with ischemic strokes having a twofold higher incidence compared to the general population [3]. Similarly, cerebral venous thrombosis occurs more frequently in cancer patients compared to the general population [4]. The incidence of ischemic stroke peaks in the six-month period before and after a cancer diagnosis, then declines after one year, whereas hemorrhagic stroke does not follow the same trend [5, 6]. Over half of patients diagnosed with both cancer and stroke have an advanced or metastatic disease [6]. Furthermore, the histology and site of cancer play a significant role. Adenocarcinomas are the most associated with ischemic stroke [6], as are lung, pancreatic, colorectal, breast, prostate, gastric, and urinary tract cancers [7]. In contrast, primary and metastatic brain tumors, hematologic neoplasms, and prostate cancer are the most frequently observed malignancies in patients with intracerebral hemorrhage [7].

Indeed, active cancer is more common in stroke patients, particularly those with cryptogenic stroke, multiple ischemic lesions on brain MRI, elevated D-dimer levels, low hemoglobin, and a history of smoking. In patients with these characteristics, cancer screening should always be considered, as stroke may be its first clinical manifestation [6, 8, 9].

The distribution of ischemic stroke subtypes in cancer patients differs significantly from that of the general population: nearly 50% of cases are cryptogenic, with a reduced proportion of cases due to large artery atherosclerosis, cardioembolism, and small vessel disease [10, 11].

Considering cancer-related molecular alterations, the frequency of cryptogenic stroke subtype, and laboratory findings in this population, the proposed mechanisms of cancer-associated stroke mainly consider an altered coagulation profile. Both cancer and the immune response trigger the production of prothrombotic molecules, abnormal platelet activation, and neutrophil extracellular trap formation; furthermore, patients with cancer show an increased prevalence of antiphospholipid antibodies [4]. Additional factors, including surgery, chemotherapy, paraneoplastic vasculitis, and immune checkpoint inhibitors, can further exacerbate the prothrombotic state [7], leading to an increased risk of venous thrombosis and ischemic stroke. Indeed, about one-third of acute ischemic strokes in patients with active cancer is related to atherosclerosis, and it represents the most common cause in those with remitting cancer [12, 13]. Notably, radiotherapy accelerates atherosclerosis on different levels [4]. Other proposed mechanisms include plaque rupture promoted by systemic inflammation and, in sporadic cases, direct tumor effects such as embolization, vascular compression, or infiltration [4]. Interestingly, new-onset atrial fibrillation has been associated with a higher risk of occult cancer, and atrial fibrillation is present in 2–5% of patients at the time of cancer diagnosis [14]. In this population, bleeding risk factors require even more careful evaluation before initiating anticoagulation than in the general population.

So far, no clinical trials have specifically addressed primary stroke prevention in high-risk cancer patients. However, anticoagulation for atrial fibrillation and venous thromboembolism prevention has proven effective in this population [15–18]. Studies comparing anticoagulants and antiplatelet agents for secondary stroke prevention in cancer patients have not demonstrated a significant difference in stroke recurrence rates between the two therapies [19]. Currently, an ongoing clinical trial is evaluating the use of edoxaban for treating coagulopathy in patients with cancer and stroke (ENCHASE Study, https://clinicaltrials.gov/study/NCT03570281?term=Edoxaban%20cancer&rank=5).

Intravenous thrombolysis (IVT) and mechanical thrombectomy (MT) are cornerstone treatments for acute ischemic stroke. For stroke patients with cancer, current international guidelines generally do not contraindicate treatment, with a few exceptions. However, these patients are treated less frequently due to various factors, primarily related to bleeding risk, frailty, pre-stroke disability, and life expectancy [20]. Previous studies have highlighted a largely unjustified exclusion of these patients from IVT and have suggested clearer inclusion and exclusion criteria for the treatment [20–22]. In this review, we summarize the most relevant evidence in this field to guide best clinical practice.

## Methods

In February 2025, we conducted a non-systematic literature search using PubMed to investigate the current evidence regarding intravenous thrombolysis (IVT) in patients with acute ischemic stroke and either active or prior cancer. Our search utilized combinations of the following keywords: “stroke,” “thrombolysis,” “IVT stroke,” or “intravenous thrombolysis,” along with “cancer,” “tumor,” “neoplasm,” or “malignancy.” We did not impose any time restrictions on the search.

No filters were applied concerning publication date or study type, and we only considered articles published in English. Additionally, we reviewed the reference lists of relevant papers to identify further sources. When pertinent articles were unavailable on PubMed, we searched other databases, including Google Scholar, the Cochrane Library, and Embase.

Given the narrative nature of our review, study selection was based on clinical relevance and included international guidelines, meta-analyses, cohort studies, and case series.

### Acute treatment for ischemic stroke in cancer patients

There is insufficient robust evidence regarding the efficacy and safety of IVT for ischemic stroke in cancer patients, as randomized clinical trials have either excluded these patients or included only a limited number of them [9]. Moreover, in emergency settings, cancer is often addressed as a single entity, despite encompassing a diverse spectrum of diseases with distinct molecular and pathological characteristics. This oversimplification, also present in clinical studies with small sample sizes, must be acknowledged along with its inherent limitations.

The 2019 American Heart Association/American Stroke Association (AHA/ASA) guidelines do not contraindicate IVT for patients with cancer, except for those with gastrointestinal and intra-axial neoplasms. IVT in these cases is considered harmful due to the increased risk of secondary bleeding (Class of recommendation III: Harm, Level of evidence C-Expert opinion). For all other cancer patients, the guidelines state that although the efficacy of IVT is not well established, treatment may be beneficial if life expectancy exceeds six months and no other contraindications exist (Class of recommendation IIb: Weak, Level of evidence C-Limited data). Among these contraindications, low platelet count, recent surgery, and systemic bleeding are the most frequently encountered [23].

Similarly, the 2021 European Stroke Organization (ESO) guidelines state that cancer does not represent an absolute contraindication to IVT, though caution is advised. The strength of recommendation is weak, and the quality of evidence is very low. Unlike AHA/ASA, ESO does not issue specific warnings for gastrointestinal or intra-axial tumors, while other contraindications remain similar [24].

In recent years, several meta-analyses have examined data from various case series and cohort studies to improve our understanding of this topic. However, the results often vary, likely due to the small sample sizes involved in these studies [9, 25–39]. Table 1 presents an overview of the structure and findings of these research efforts. Here, we discuss their aggregated results as synthesized in two recent systematic reviews and meta-analyses, published after the release of at least the first one of the abovementioned international guidelines.

**Table 1:** **Studies on intravenous thrombolysis (IVT) in patients with acute ischemic stroke and cancer**. CE: cardioembolism; ICA: internal carotid artery; ICH: intracerebral hemorrhage; LAA: large artery atherosclerosis; MCA: middle cerebral artery; mRS: modified Rankin scale; MT: mechanical thrombectomy; NIHSS: National institutes of health stroke scale; N/A: not applicable; PACI: partial anterior circulation infarction; POCI: posterior circulation infarction; sICH: symptomatic intracerebral hemorrhage; SVO: small-vessel occlusion; TACI: total anterior circulation infarction; TOAST: trial of acute stroke treatment classification; UND: undetermined etiology; vasc. Terr.: vascular territory

Authors and publication year	Nationality	Study and population	Cases	Controls	Stroke characteristics (vascular territory and TOAST classification)	Outcomes
Graber et al., 2012 [26]	USA	Single-center case series; patients with ischemic stroke receiving IVT	6 patients with active cancer	None	*Vasc. Terr.*: N/A. *TOAST*: 1 LAA, 1 CE, 4 UND.	No ICH following IVT; one extractranial bleeding at tumor site during IVT administration; 4/6 improved within 72 h; three died within one month
Cappellari et al., 2013 [32]	Italy	Single-center case series; patients with ischemic stroke receiving IVT	11 patients with active, non-metastatic cancer	None	*Vasc. Terr.*: 5 TACI, 4 PACI, 2 POCI. *TOAST*: 2 LAA, 5 CE, 3 UND.	No severe hemorrhagic complications, no major localized or systemic bleeding following IVT; reduced NIHSS at 7 days for all patients; 90-day mRS 0–2 in 8 patients
Murthy et al., 2013 [38]	USA	Multi-center cohort study; patients with ischemic stroke receiving IVT (subgroup analysis, whole study including also patients treated with MT + IVT)	614 patients with cancer	25,196	*Vasc. Terr.*: N/A. *TOAST*: N/A.	No difference in in-hospital mortality, ICH; higher in-hospital mortality and lower home discharge for solid and metastatic tumors
Sobolewski et al., 2015 [30]	Poland	Multi-center cohort study; patients with ischemic stroke receiving IVT	40 patients with cancer	495	*Vasc. Terr.*: N/A. *TOAST*: 19 LAA, 8 CE, 5 SVO, 8 UND.	No difference in sICH, hemorrhagic transformation, 90-day disability (mRS 0–2), 90-day mortality
Geraldes et al., 2017 [29]	Spain	Single-center cohort study; patients with ischemic stroke receiving IVT	7 patients with active cancer	20	*Vasc. Terr.*: N/A. *TOAST*: 2 CE, 7 UND.	No difference in direct procedure complications, hemorrhagic transformation; one case with systemic hemorrhage and one control with severe ICH
Nam et al., 2017 [25]	Korea	Multi-center case series; patients with ischemic stroke receiving IVT	12 patients with active cancer	None	*Vasc. Terr.*: N/A. *TOAST*: 4 LAA, 2 CE, 6 UND.	Poor outcomes (early neurological deterioration, hemorrhagic transformation, in-hospital mortality, mRS 3–6); worse mRS for cryptogenic cases
Selvik et al., 2018 [9]	Norway	Single-center cohort study; patients with ischemic stroke receiving IVT	5 patients with active cancer	266	*Vasc. Terr.*: N/A. *TOAST*: 2 CE, 1 SVO, 2 UND.	No adverse event from IVT in any case patient. No comparison with “controls” except for the number of treatments (16% of controls, 6% of cases)
Seo et al., 2018 [34]	Korea	Multi-center cohort study; patients with ischemic stroke receiving IVT	13 patients with active cancer	148	*Vasc. Terr.*: N/A. *TOAST*: 2 LAA, 4 CE, 7 UND.	Higher incidence of hemorrhagic transformation, sICH, and mortality
Sallustio et al., 2019 [27]	Italy	Single-center cohort study; patients with ischemic stroke receiving IVT (subgroup analysis, whole study including also patients treated with MT and MT + IVT)	11 patients with active cancer	11	*Vasc. Terr.*: 11 anterior circulation strokes caused by MCA occlusion, about half in the M1 segment. Tandem ICA-MCA occlusion in a few patients. *TOAST*: N/A.	No difference in sICH, 90-day disability (mRS 0–2), 90-day mortality
Weeda et al., 2019 [28]	USA	Multi-center cohort study; patients with ischemic stroke receiving IVT	416 patients with active cancer	13,577	*Vasc. Terr.*: N/A. *TOAST*: N/A.	No difference in in-hospital mortality; increased ICH incidence and length of stay
Merlino et al., 2021 [36]	Italy	Single-center cohort study; patients with ischemic stroke receiving IVT (subgroup analysis, whole study including also patients treated with MT and MT + IVT)	46 patients with active cancer	33 patients with remote cancer and 361 patients with no history of cancer	*Vasc. Terr.*: N/A. *TOAST*: 5 LAA, 21 CE, 3 SVO, 17 UND.	No difference in neurological improvement, sICH, 90-day disability, and in-hospital mortality; increased 90-day all-cause (and not stroke-related) mortality for active cancer patients; no worse outcome for metastatic patients
Owusu-Guha et al., 2021 [31]	USA	Multi-center cohort study; patients with ischemic stroke receiving IVT	18,781 patients with cancer	146,429	*Vasc. Terr.*: N/A. *TOAST*: N/A.	No difference in intracranial bleeding, all-cause bleeding, in-hospital mortality, 30-day readmission rate; increased 90-day readmission rate
Yoo et al., 2021 [33]	Korea	Multi-center cohort study; patients with ischemic stroke receiving IVT	62 patients with active cancer	78 patients with non-active cancer, 1198 patients with no history of cancer	*Vasc. Terr.*: 33 MCA occlusions and 9 ICA occlusions. 4 POCI. *TOAST*: N/A, but 40 patients with determined and 22 patients with UND.	No difference in 24-hour neurological improvement, ICH; poorer 90-day functional outcome (mRS 0–2) and 6-month survival in the active cancer population; worse outcome for patients with undetermined stroke etiology
Mosconi et al., 2022 [37]	Italy	Single-center cohort study; patients with ischemic stroke receiving IVT	14 patients with active cancer	248	*Vasc. Terr.*: N/A. *TOAST*: N/A.	No difference in sICH, 90-day disability (mRS 0–2), 90-day mortality. Severe extracranial bleeding at the tumor site in 3 cases (21.4%)

In 2020, Huang and colleagues published a systematic review and meta-analysis of 15 studies, concluding that cancer patients do not experience worse outcomes following IVT compared to non-cancer patients in terms of efficacy (evaluated as functional independence with modified Rankin Scale 0–2 at 3 months) and safety (hemorrhagic transformation, symptomatic intracranial hemorrhage, major bleeding, in-hospital mortality, and 3-months mortality). Cancer populations were heterogeneous in terms of cancer type [40]. Only one study specifically examined patients with stroke and primary brain tumors. Independent of treatment, it reported higher in-hospital mortality and intracranial hemorrhage compared to those with non-brain tumors; however, IVT was found to be safe, as it did not increase the risk of intracranial hemorrhage in this population [39]. Additionally, clinical outcomes were similar between patients with gastrointestinal tumors and patients with other malignancies, suggesting that this should not be considered an absolute contraindication to IVT. One study compared solid, metastatic, and liquid tumors, finding no difference in intracranial hemorrhage following IVT in the three groups, but better home discharge rates and lower in-hospital mortality for the latter [38].

More recently, in 2023, Mosconi and colleagues conducted a systematic review of the literature on this topic. From the meta-analysis of the selected 11 cohort studies, they reached similar conclusions. More specifically, the meta-analysis included 19.927 acute stroke patients with active cancer and 183.359 without. IVT did not worsen clinical outcomes in patients with active cancer, as assessed by rates of intracerebral hemorrhage, mortality, and functional independence (modified Rankin Scale 0–2) at the end of the scheduled follow-up period [41].

Interestingly, only two studies (included in Table 1 and analyzed in the aforementioned meta-analyses) reported poorer outcomes in cancer patients treated with IVT compared to non-cancer stroke patients, and both were conducted in the Republic of Korea [25, 34]. Further analyses exploring genetic background and cancer types in these populations could provide valuable insights. Notably, another finding that warrants attention is that patients with metastatic disease did not show worse responses to IVT compared to those with non-metastatic cancer, as demonstrated by one retrospective study [36].

Taken together, these findings support the use of IVT in cancer patients with stroke, raising questions about the AHA/ASA contraindications for gastrointestinal and intra-axial tumors. Nonetheless, the overall evidence remains weak, and clinicians are often hesitant to administer IVT in this population. Furthermore, cancer patients more frequently present other contraindications to IVT, primarily low platelet count, but also recent surgery, anticoagulation, and systemic bleeding. Notably, systemic bleeding is the first symptom in approximately 70% of gastrointestinal malignancies [42, 43].

### Outcomes following stroke in cancer patients

Cancer patients who experience ischemic stroke tend to have a worse prognosis in terms of neurological deterioration, mortality, and recurrence rate compared to the general population, regardless of stroke treatment [44–48]. This is largely influenced by their tumor– especially in the presence of metastases– but also by stroke severity, hemorrhagic transformation, and elevated levels of D-dimer and C-reactive protein [49, 50]. Stroke recurrence within one year is as high as 28% in this population, compared to 13% in the general population [51].

To date, no study has directly compared the outcomes of cancer patients with acute ischemic stroke treated with IVT versus those not receiving IVT. Available evidence primarily derives from observational studies and registries that assess the safety and efficacy of IVT in cancer patients without a control group of untreated individuals. Given the potential benefits of IVT in eligible cancer patients with stroke, timely treatment appears crucial. However, further studies are essential to evaluate this comparison directly.

## Discussion

The use of IVT in cancer patients with ischemic stroke is reasonable in terms of safety and efficacy, provided that individual risk factors are carefully assessed.

Although IVT should not be denied solely based on a cancer diagnosis, treating physicians must pay particular attention to exclude contraindications in this fragile population and to follow their clinical course. Here are some points to consider:

- Coagulation abnormalities: Unlike the general stroke population, cancer patients more frequently present with thrombocytopenia, disseminated intravascular coagulation, or altered coagulative profile in hematologic malignancies or in association with solid tumors and immune system response, as previously discussed. While current guidelines suggest that IVT may be administered without waiting for laboratory results in patients without suspected bleeding disorders [23, 24], this assumption is less reliable in oncologic patients. Recent blood examinations, if available, should be reviewed; otherwise, physicians must consider the possibility of waiting for those performed in the emergency setting. Also, a thorough history is critical to identify active bleeding episodes, even if minor, that could indicate an increased hemorrhagic risk.
- Recent surgery: Receiving a cancer diagnosis is often followed by major surgery. Even if this is not explicitly reported by the patient or caregiver or mentioned in the clinical history, physicians must investigate thoroughly to exclude it.
- Concomitant medications: Many cancer patients receive anticoagulants (for venous thromboembolism prophylaxis or treatment), and several chemotherapeutic and targeted therapies may exacerbate the risk of hemorrhagic complications. Among these, some molecules specifically target vascular endothelial growth factor (VEGF) such as bevacizumab, but also tyrosine kinase inhibitors can impact hemostasis by acting on platelets [52, 53]. On the other side, some chemotherapeutic drugs can induce thrombocytopenia, mainly platinum derivatives, docetaxel, and gemcitabine [54]. An accurate pharmacological assessment is thus crucial.
- Gastrointestinal and intra-axial cancer: In the 2019 AHA/ASA guidelines, these are listed as relative contraindications to IVT, primarily due to concerns about an increased risk of bleeding. Intra-axial tumors—particularly high-grade gliomas and metastases—are often highly vascularized and infiltrative, potentially heightening the risk of intracerebral hemorrhage after thrombolysis. Similarly, advanced gastrointestinal cancers may predispose to mucosal friability or ongoing bleeding, raising concerns about major extracranial hemorrhagic complications. These cautions, however, are largely precautionary and supported by limited clinical evidence. This may help explain the differing stance of the ESO 2021 guidelines, which recommend a more individualized approach in such cases. Moreover, recent meta-analyses suggest that IVT outcomes in these subgroups are not significantly worse than in other cancer patients, provided that overt gastrointestinal bleeding is absent [40]. Following the ESO guidelines, these patients should not be denied IVT a priori; rather, they should be evaluated on an individual basis. A similar, though more cautious, approach may be needed for patients with intracranial neoplasms, acknowledging the current uncertainty in risk estimation. Even in the acute setting, for a complete evaluation of the case, physicians must remember that patients with melanoma, lung, and breast cancer have the highest risk of having brain metastases. Checking the CT scan with this information in mind can be helpful.
- Stroke pattern and infarct burden: Cancer-related stroke often presents with multiple infarcts or large ischemic areas, both of which independently increase the likelihood of hemorrhagic transformation. Physicians should be particularly cautious in patients with suspected extensive ischemia (clinical severity) or infarcts in critical locations.
- Cautious management: Continuous monitoring during and after IVT administration is essential to promptly detect signs of intracranial or systemic hemorrhage, which is particularly crucial in these patients due to the potentially increased hemorrhagic risk. Moreover, the management of cancer patients with stroke should involve a dedicated team of neurologists, neuroradiologists, oncologists, hematologists, and intensive care specialists to ensure an integrated and personalized approach.

**Fig. 1:**
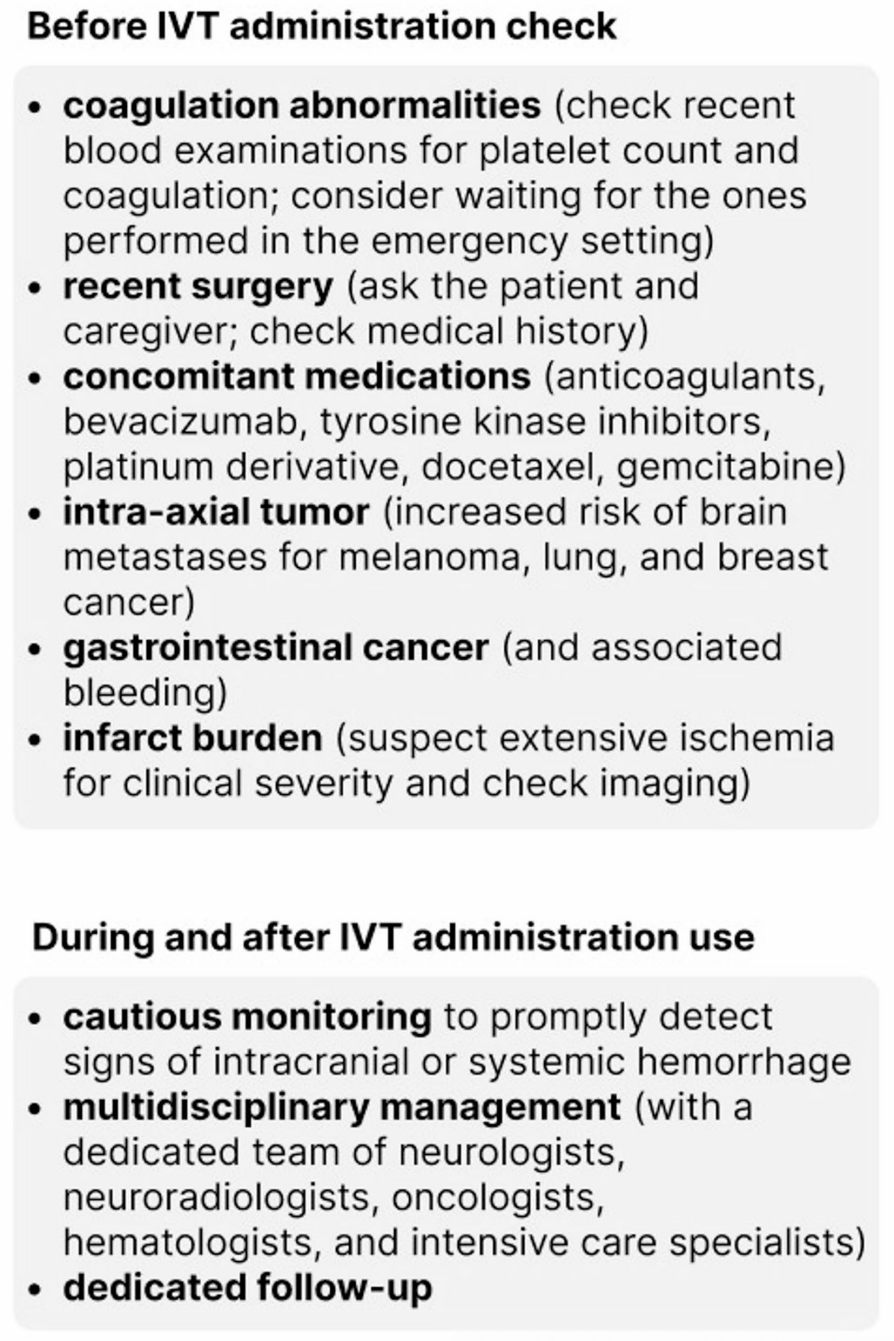
**Checklist for intravenous thrombolysis (IVT) administration in cancer patients**. A practical reminder for clinicians in emergency settings. IVT: intravenous thrombolysis

Figure 1 summarizes the key points discussed above in a checklist format for clinical practice.

Despite the growing body of evidence supporting IVT in cancer patients with acute ischemic stroke, large, prospectively designed studies are necessary to better define the risks and benefits in this complex and heterogeneous population. Ethical considerations may preclude the use of randomized controlled trials, given the proven efficacy and safety to date; however, well-structured observational studies could still provide valuable insights.

Future research should also aim to stratify patients based on tumor histology and molecular characteristics, as the mechanisms underlying cancer-associated coagulopathy vary widely. For instance, adenocarcinomas are strongly linked to hypercoagulability, while hematologic malignancies may predispose more to thrombocytopenia and hemorrhagic complications. Understanding these distinctions could help refine treatment algorithms.

Another important gap in the existing literature is that of studies directly comparing cancer patients with stroke who receive IVT versus those who do not. Such studies would be valuable in clarifying the impact of intravenous reperfusion therapy on both favorable and unfavorable outcomes. Once more, randomized trials pose ethical challenges, but well-designed prospective and retrospective studies could provide crucial insights to guide future clinical decision-making.

Current literature on this topic focuses on alteplase as an IVT agent; however, in other settings, tenecteplase has shown a comparable or even superior safety profile [55–58]. Its greater fibrin specificity and lower likelihood of fibrinogen depletion might reduce bleeding risk, which is particularly relevant given the complexity of cancer patients [59, 60]. Further studies are advisable on this alternative thrombolysis agent.

From a practical standpoint, better integration of electronic health records into acute stroke care could significantly enhance decision-making. Most patients with stroke arrive at the emergency department unable to communicate effectively, and obtaining a rapid, accurate history is challenging. A system that automatically retrieves key medical data– including recent laboratory values, active treatments, prior surgeries, and known diagnoses– could be transformative, reducing uncertainty in acute therapeutic decisions.

Although discussing MT is beyond the scope of this review, during our literature review, we came across studies on its role in cancer patients. So far, they indicate that recanalization outcomes in cancer patients are comparable to those in the general stroke population. Moreover, the incidence of intracranial hemorrhage following MT does not appear to be increased in cancer patients [61–65]. These findings reinforce the notion that acute reperfusion therapies should not be systematically withheld in cancer patients with ischemic stroke, even though further research is needed to refine treatment strategies in this complex population.

As cancer survival rates continue to improve, optimizing stroke care in this population will become increasingly important. A more refined, evidence-based approach is needed, one that carefully balances the risk of hemorrhage with the potential to preserve neurological function and quality of life. Every therapeutic decision should be made based on a comprehensive, individualized assessment, ideally conducted by a multidisciplinary team of specialists. The goal is to deliver the best possible short- and long-term outcomes for cancer patients.

## Conclusions

Cancer patients experience ischemic stroke more frequently than the general population, often with distinctive features such as cryptogenic etiology and multiple lesions. Their management represents a complex challenge, requiring a careful balance between maximizing the benefits of acute reperfusion therapies and minimizing hemorrhagic risks that intrinsically depend on their condition and its treatment. Current evidence, although limited, supports the safety and efficacy of IVT and MT in this population, suggesting that these treatments should not be systematically denied. However, specific considerations (i.e., coagulation abnormalities, recent surgery, concomitant medications, tumor site, and tumor type) should always be carefully evaluated on an individual basis.

Despite progress in understanding the pathophysiology and best treatment practice of cancer-associated stroke, knowledge gaps remain. Future research should focus on refining risk stratification models and incorporating tumor biology into treatment decision-making.

As cancer survival rates improve, stroke management in this population will become increasingly relevant. A multidisciplinary approach is crucial to optimizing care with the ultimate goal of providing the best possible neurological and oncological outcomes, ensuring that cancer patients receive timely and appropriate stroke treatment.

## Data Availability

No datasets were generated or analysed during the current study.
